# Allele frequency changes due to hitch-hiking in genomic selection programs

**DOI:** 10.1186/1297-9686-46-8

**Published:** 2014-02-04

**Authors:** Huiming Liu, Anders C Sørensen, Theo HE Meuwissen, Peer Berg

**Affiliations:** 1 Center for Quantitative Genetics and Genomics, Department of Molecular Biology and Genetics, Aarhus University, P. O. Box 50, 8830 Tjele, Denmark; 2 Department of Animal and Aquacultural Sciences, Norwegian University of Life Sciences, P. O. Box 5003, 1432 Ås, Norway; 3 Nordic Genetic Resource Center, P. O. Box 115, 1431 Ås, Norway

## Abstract

**Background:**

Genomic selection makes it possible to reduce pedigree-based inbreeding over best linear unbiased prediction (BLUP) by increasing emphasis on own rather than family information. However, pedigree inbreeding might not accurately reflect loss of genetic variation and the true level of inbreeding due to changes in allele frequencies and hitch-hiking. This study aimed at understanding the impact of using long-term genomic selection on changes in allele frequencies, genetic variation and level of inbreeding.

**Methods:**

Selection was performed in simulated scenarios with a population of 400 animals for 25 consecutive generations. Six genetic models were considered with different heritabilities and numbers of QTL (quantitative trait loci) affecting the trait. Four selection criteria were used, including selection on own phenotype and on estimated breeding values (EBV) derived using phenotype-BLUP, genomic BLUP and Bayesian Lasso. Changes in allele frequencies at QTL, markers and linked neutral loci were investigated for the different selection criteria and different scenarios, along with the loss of favourable alleles and the rate of inbreeding measured by pedigree and runs of homozygosity.

**Results:**

For each selection criterion, hitch-hiking in the vicinity of the QTL appeared more extensive when accuracy of selection was higher and the number of QTL was lower. When inbreeding was measured by pedigree information, selection on genomic BLUP EBV resulted in lower levels of inbreeding than selection on phenotype BLUP EBV, but this did not always apply when inbreeding was measured by runs of homozygosity. Compared to genomic BLUP, selection on EBV from Bayesian Lasso led to less genetic drift, reduced loss of favourable alleles and more effectively controlled the rate of both pedigree and genomic inbreeding in all simulated scenarios. In addition, selection on EBV from Bayesian Lasso showed a higher selection differential for mendelian sampling terms than selection on genomic BLUP EBV.

**Conclusions:**

Neutral variation can be shaped to a great extent by the hitch-hiking effects associated with selection, rather than just by genetic drift. When implementing long-term genomic selection, strategies for genomic control of inbreeding are essential, due to a considerable hitch-hiking effect, regardless of the method that is used for prediction of EBV.

## Background

Genetic improvement in livestock is driven by increasing the frequency of favourable alleles at loci that affect the traits of interest in populations [1]. The magnitude of these increases is mainly determined by the allele substitution effects and allele frequency at these loci, along with the intensity and accuracy of artificial selection [2]. Genomic selection (GS) provides opportunities to enhance the accuracy of prediction of breeding values. Conventional selection methods exploit phenotypes of the individual and/or of its relatives’, e.g. using best linear unbiased prediction (BLUP) [3,4], whereas GS combines marker data with phenotypic and pedigree data (when available), which increases the accuracy of prediction. In addition, marker data allow accurate estimation of mendelian sampling effects in GS, allowing more accurate within-family selection, which leads to a lower level of pedigree-based inbreeding in GS compared to BLUP [5,6].

Pedigree-based inbreeding, however, might not reflect the true level of inbreeding. First, pedigree inbreeding is an expectation of the proportion of the genome that is autozygous (homozygosity caused by two identity-by-descent (IBD) genomic segments) but there is much variation around this expectation due to the stochastic nature of recombination [7]. For instance, the percentage of the genome that is autozygous among progeny of first cousins is 6.25% on average, with a standard deviation of 2.4% [7-9]. Second, the level of inbreeding greatly depends on the generation that is considered as the founder generation. Animals in the defined founder population are considered to be unrelated, although, in reality they are related. Third, pedigree inbreeding assumes that there are no systematic changes in allele frequencies due to selection, which means that the loci are expected to be neutral. This assumption will not hold if selection is performed on a trait that is controlled by a few QTL with large effect or a complex trait that is controlled by a large number of QTL and the size of the genome is limited. With selection, inbreeding at the QTL arises from selectively increasing the frequency of favourable alleles towards homozygosity, which may also give rise to a footprint of selection surrounding the QTL due to hitch-hiking [10]. As a result of hitch-hiking, selection will inevitably act on closely linked neutral loci and force them towards fixation, which may increase both allozygosity (homozygosity produced by alleles that are identical by state) and autozygosity, and thus will raise the level of inbreeding in the region surrounding the QTL [11-13]. Therefore, pedigree inbreeding substantially underestimates the loss of genetic variance, in particular in the region that contains a QTL with a large effect. Pedersen et al. [13] found that for selection based on BLUP EBV, the rate of genomic inbreeding at all linked neutral loci across a chromosome that contains a major QTL was significantly higher than the rate of pedigree inbreeding. This indicates that, due to hitch-hiking effects, there are no neutral loci on a chromosome that contains a QTL.

The development of technologies for typing dense marker genotypes provides opportunities to more precisely measure the fraction of the genome that loses genetic variability during selection. Dense marker genotypes can also be used to scan the genome of animals for runs of homozygosity (ROH). Runs of homozygosity in an individual result from the inbreeding to a common ancestor by inheriting chromosome segments that are IBD from both parents [14]. The longer (shorter) such segments are, the more recent (ancient) the relatedness is. Therefore, ROH is expected to provide a more accurate measure of relatedness and may be a better indicator for the true level of inbreeding than pedigree-based relatedness.

In addition, previous studies have revealed that, when performing selection for many generations, GS increases the risk of losing favourable QTL alleles compared to phenotypic selection [15], in particular in the first few generations. Some of these alleles are rare and unavoidably lost due to low linkage disequilibrium (LD) with any marker [16]. The remaining favourable QTL alleles are essential to maintain long-term genetic variance and response to selection. However, a systematic comparison of the loss of favourable and rare alleles between genomic and conventional selection methods as selection proceeds is lacking.

The main purpose of this study was to evaluate the impact of long-term selection on changes in allele frequencies due to hitch-hiking and inbreeding. To achieve this, we first monitored (i) the fixation and loss of favourable alleles, (ii) the maintained genetic variance, and (iii) the accuracy of selection when employing genomic or conventional selection methods. Second, to better understand the effect of hitch-hiking, we explored the reduction of heterozygosity at loci that are closely linked to QTL for different selection methods. Third, we compared the overall level of inbreeding measured by ROH and pedigree. Fourth, we assessed to what extent changes in allele frequencies and inbreeding are affected by genetic architecture, i.e. heritability and the number of QTL.

## Methods

### Simulation design

We compared GS on genomic EBV derived with two commonly used approaches: (i) genomic BLUP (GBLUP) and (ii) Bayesian Lasso (BL), with two conventional selection methods, (iii) phenotypic selection (PS) and (iv) selection on EBV derived using phenotype-based BLUP. A detailed description of these models is in the section “Selection criteria”. The comparison was performed for all combinations of two levels of heritability and three numbers of QTL affecting the trait (Table 1). Apart from changes in allele frequencies, inbreeding coefficients based on pedigree, genetic variance, and accuracy of selection were followed for 25 generations.

**Table 1:** **Summary of scenarios with respect to heritability, number of QTL (nQTL) and initial variance contributed by each QTL**$\left(\sigma_{\text{qtl}}^{2}\right)$

**Scenarios**	**Heritability**	**nQTL**	$\sigma_{\mathrm{qtl}}^{2}$ ^*^
*4QTL_h5*^†^	0.05	4	1.25e-2
*4QTL_h25*	0.25	4	6.25e-3
*40QTL_h5*	0.05	40	1.25e-3
*40QTL_h25*	0.25	40	6.25e-4
*100QTL_h5*	0.05	100	1.25e-4
*100QTL_h25*	0.25	100	6.25e-5

^*^Each QTL was assumed to contribute equal variance; ^†^scenarios are represented by xQTL_hy where x is the number of QTL and y is the value of heritability multiplied by 100.

### Genome structure

Initially a historical population with an effective size of 200 (N_e_ = 200) was simulated using QMSim [17]. The 200 animals were mated at random for 2000 discrete generations, with an equal sex ratio and without selection or migration. The simulated genome consisted of five 1 Morgan chromosomes. Ten thousand loci were positioned equally across each chromosome, resulting in 50 000 loci across the genome. In generation 0, all loci were set to be bi-allelic with allele frequencies equal to 0.5 and alleles coded as “1” and “2”. Recurrent mutations were simulated at a rate of 2.5 × 10^−5^ per locus per meiosis in the subsequent generations. Recombinations per chromosome were sampled from a Poisson distribution with a mean equal to the length of the chromosome in Morgan and were randomly placed along the chromosome assuming a uniform distribution. Generation 2000 was used as the base population (G_0_). In G_0_, the average linkage disequilibrium (LD) (±SD) between neighboring loci was r^2^ = 0.26 (±0.34) and the allele frequency distribution followed a U-shaped distribution, with 30.6% of the loci fixed. For the analysis in G_0_ and onwards, markers and QTL were chosen among all segregating loci and the simulations were programmed using R [18].

For chromosomes 1 to 4, among all simulated loci, every second locus was used as a potential marker. The remaining loci were used as potential QTL. Potential markers with a minor allele frequency (MAF) lower than 0.05 in G_0_ were discarded. From the 5000 potential QTL, a specified number of QTL were selected, depending on the scenario (Table 1). For each QTL, the allele coded as “1” was used as the favorable allele. Potential QTL that had a frequency of 0.01, 0.1 or 0.3 (±2% of defined frequency) for allele “1” were used as QTL. These three sets of low initial allele frequencies were chosen in order to be as far as possible from fixation and to observe the loss of favorable alleles with different initial allele frequencies. Rare alleles were considered as those having a favorable allele frequency of 0.01. The positions of QTL were varied per replicate, but the same base population was used for all four selection criteria within each replicate. Potential QTL that were not used as QTL (with MAF > 0.01) were used as linked neutral loci (LN). These loci were assumed to have no effects on the trait and were therefore not used for selection. No QTL were simulated on chromosome 5 and 2000 loci with a MAF > 0.01 were randomly chosen from the 10 000 simulated loci on this chromosome and used as selectively neutral loci (SN). In descendant generations, genotypes with respect to QTL, markers, LN and SN were sampled according to the mendelian inheritance principles allowing for recombination. The simulation of recombination was the same as for the historical population.

### Trait simulation

The considered traits were standardized to have a mean of 0 and variance equal to the defined heritability for animals in G_1_ (Table 1). Generations 1 to 25 were simulated without mutations. Each QTL was assumed to have the same additive genetic variance, so the allele substitution effect at QTL *j* was set equal to: $\alpha_{j}=\sqrt{\frac{h^{2}}{\;2p_{j}\left(1-p_{j}\right)n}}$, where *h*^2^ is the heritability, *n* is the number of QTL, and *p*_*j*_ is the frequency of allele “1” of QTL *i*[2]. No dominance or epistatic effects were included. The true breeding value (TBV) for each animal was obtained by summing the allelic effects at each QTL. The environmental terms were drawn from a random normal distribution *N*(0, 1 − h^2^) and were added to the TBV to obtain the phenotypic record of each animal. Environmental variance was kept constant through the simulations, regardless of changes in additive genetic variance. The methods for simulating TBV and phenotypic records were identical in each generation.

Selection on the different criteria started from G_1_ and was continued for 25 generations. In each generation, the best 50 males and 50 females among 400 candidates were selected based on the selection criterion. Selected individuals were randomly mated and each pair produced eight offspring with equal sex ratio.

### Selection criteria

Breeding values were estimated for all individuals from G_1_ onwards using the four methods of interest. For PS, selection was simply on the individual’s own phenotype. For BLUP, the pedigree and phenotype for G_t_ and G_t-1_ were assumed to be known. For GBLUP and BL, the marker genotype and phenotype for G_t_ and G_t-1_ were assumed to be known. The use of information of only the two generations was chosen in order to allow a fair comparison between different selection criteria and to reduce computation time.

The following general structure of models [19] was used to predict EBV with BLUP and GBLUP for individuals in generation G_t_:

y=1μ+Zg+e

where **y** is the vector of phenotypic records from G_t-1_ and G_t_, *μ* is the overall mean, **1** is a vector of 1’s, **Z** is an incidence matrix for allocating phenotypes to breeding values, **g** is a vector of breeding values to be estimated, and **e** is a vector of residuals assumed $N\left(0,I\sigma_{e}^{2}\right)$, where **I** is an identity matrix.

#### BLUP method

The true breeding values for BLUP were assumed to follow a normal distribution $N\left(0,\;A\sigma_{a}^{2}\right)$, where **A** is the numerator relationship matrix based on the pedigree. BLUP was performed by solving the mixed model equations for the animal model given the inverse of the numerator relationship matrix, **A**^**−1**^, which was calculated based on individuals in G_t_ and their sires and dams in G_t-1._

#### GBLUP method

With GBLUP, true breeding values were assumed to follow a normal distribution $N\left(0,G\sigma_{a}^{2}\right)$, where **G** is the genetic relationship matrix based on the marker data [20]. GBLUP was performed by solving the mixed model equations for the animal model given **G**.

#### Bayesian Lasso method

A BL model was built according to the description in [21,22]. The breeding value g_*i*_ for individual *i* was defined as a parametric linear regression on marker covariates *x*_*ij*_ of the form $g_{i}=\sum_{j=1}^{p}x_{\mathrm{ij}}\beta_{j}$, such that $y_{i}=\mu +\sum_{j=1}^{p}x_{\mathrm{ij}}\beta_{j}+e_{i}$, where *y*_*i*_ is the phenotypic record of an individual from G_t-1_ or G_t_, *μ* is the intercept, and {*β*_*j*_}_*j*=1_^*p*^ are the marker effects (*j* =1,2,…., p markers). Gaussian assumptions for model residuals were used, i.e. the joint distribution of model residuals was assumed to follow $N\left(0,\sigma_{e}^{2}\right)$. The likelihood function yields:

$$p\left(y\left|\mu ,g,\sigma_{e}^{2}\right)\right)=\prod_{i=1}^{n}N\left(y_{i}|\mu +\sum_{j=1}^{p}{x_{i}}_{j}\;\beta_{j},\sigma_{e}^{2}\right),$$

where $N\left(\left(y_{i}\right|\mu +\sum_{j=1}^{p}x_{\mathrm{ij}}\;\beta_{j},\sigma_{e}^{2}\right)$ is a normal density for random variable *y*_*i*_ centered at $\mu +\sum_{j=1}^{p}{x_{i}}_{j}\;\beta_{j}$ and with variance $\sigma_{e}^{2}$. The BL assigns a double exponential distribution to all marker effects, conditional on a regularization parameter **λ**, centered at zero and with marker-specific variance: $p\left(\left(\beta_{j}\right|0,\frac{\lambda }{\sigma_{e}^{2}}\right)$. The prior distribution for the residual variance was an inverse-chi-square distribution with 4 degrees of freedom and scale parameter 1. The rate and shape parameters for **λ** were set to 1 × 10^−4^ and 0.6, respectively, following the guideline of [23]. The marker effects were estimated using the BL described in [24], as implemented in the BLR package of R [25]. Further details on the model and algorithms can be found in [21,22]. The Gibbs sampler was run for 1500 iterations and the first 500 iterations were discarded as burn-in.

### Data analysis

The summary statistics for each of the scenarios were based on 100 replicated simulations. Allele frequency changes at all loci, genetic variance, accuracy of selection and inbreeding under all selection criteria were calculated for each generation and were used for comparisons. A favorable allele was considered fixed when *p* = 1 and lost when *p* = 0. Allele frequency changes at all loci in generation t (Δ*p*_*t*_) were scaled by a factor $\sqrt{p_{t-1}*\left(1-p_{t-1}\right)}$ in order to standardize Δ*p*_*t*_ relative to the standard deviation of the frequencies. The Δ*p*_*t*_ of fixed and lost alleles were not used in the analysis. Changes in allele frequencies at LN and SN were compared to quantify the hitch-hiking effect, i.e. whether the evolution of a selected locus (QTL) may alter the dynamics of many closely linked loci in comparison to neutral loci [26]. Allele frequency changes resulting from sampling were random, in the sense that their directions were unpredictable, but their magnitude can be predicted in terms of the variance of the changes [2]. Therefore, the variance rather than the mean of allele frequency changes was used for analysis. The level of hitch-hiking for LN was measured by dividing the variance of allele frequency changes for LN by the variance of allele frequency changes for SN $\left(\frac{\sigma^{2\;}\left(\Delta p_{\mathrm{LN}}\right)}{\sigma^{2}\left(\Delta p_{\mathrm{SN}}\right)}\right)$. The numerator *σ*^2^(Δ*p*_*LN*_) is the measure of drift due to indirect selection and sampling, whereas the denominator *σ*^2^(Δ*p*_*SN*_) is the measure of drift only due to sampling. Values greater than 1 indicate a hitch-hiking effect.

Genetic variance was calculated from the variance of TBV within generation. Accuracy was calculated as the correlation between the selection criteria, i.e. phenotype or estimated (G)EBV, with TBV for the G_t_ animals.

Pedigree-based inbreeding coefficients in G_t_, F_ped_, were estimated with the inbreeding function in the GeneticsPed package [27] of R, using the algorithm by Meuwissen and Luo [28] and all pedigree information from G_0_ to G_t_. Individuals in G_0_ were assumed to be unrelated. Runs of homozygosity (ROH) were detected for each animal by PLINK [29], using a sliding window of 10, 25 or 50 consecutive markers across the genome. No heterozygous marker genotype was allowed within a given window. A map file was created from the location of all loci on the chromosome and the unit was directly converted from centi-Morgan to base-pairs (1 cM = 10^6^ bp). If the gap between two consecutive homozygous markers was greater than 1 Mb, the ROH was split into two. Inbreeding coefficients estimated by ROH (F_ROH_) were calculated for each animal as the fraction of the genome covered by markers involved in ROH. The rate of inbreeding (*ΔF*_*t*_) for F_ped_ or F_ROH_ was calculated as $\Delta F_{t}=1-\sqrt[{t}]{\frac{1-F_{t}}{1-F_{0}}}$, which was derived from the equation in [2], where F_*t*_ and F_0_ are the inbreeding coefficients in G_t_ and in the base population (F_0_). In addition, the mendelian selection differential was calculated by the method of Pedersen et al. [13]. The mendelian sampling term was calculated as the difference between an animal’s TBV and the mean TBV of its parents. The mendelian selection differential was then calculated as the difference between the mean mendelian sampling term of the selected animals and that of all animals within a generation. Comparisons of rates of inbreeding and mendelian selection differentials between scenarios were performed using Tukey’s HSD (honestly significant difference) test (p < 0.05).

## Results

### Changes in the frequency of favourable QTL alleles

**Figure 1:**
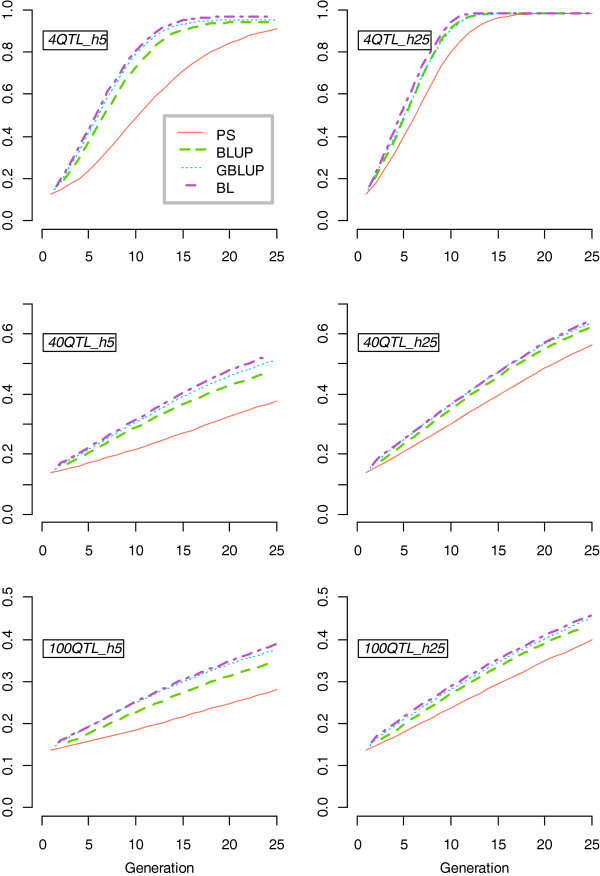
**Favourable QTL allele frequencies (p) across 25 generations.** Note that the scale of the y-axis is not the same for scenarios with different numbers of QTL.

**Figure 2:**
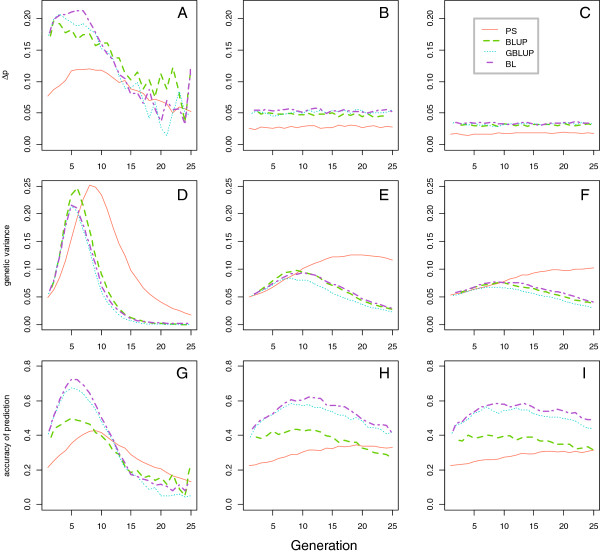
**Favourable QTL allele changes (∆p), genetic variance and accuracy of prediction across 25 generations in the *h5 *scenarios (h**^**2**^ **= 0.05). A-C**: favourable QTL allele frequency changes (∆p) in *4QTL_h5*, *40QTL_h5*, *100QTL_h5* scenarios; **D-F**: genetic variance among selection candidates in each generation in *4QTL_h5*, *40QTL_h5*, *100QTL_h5* scenarios; **G-I**: accuracy of predicting breeding values in *4QTL_h5*, *40QTL_h5*, *100QTL_h5* scenarios.

Figure 1 shows that in general, favourable QTL allele frequencies (p) increased significantly faster when the number of QTL was lower and heritability was higher. Genomic information used by BL and GBLUP increased the average frequency of favourable QTL alleles, p, only marginally compared to BLUP and PS at higher heritability, while the differences in p between all selection criteria were more pronounced at lower heritability. Results from the *h5* scenarios were focused on to demonstrate these differences (Figure 2).

The difference in favourable QTL allele frequencies, p, between selection criteria was largest in the *4QTL_h5* scenario, where BL on average fixed all favourable alleles approximately 10 generations earlier than PS (Figure 1). In the *4QTL_h5* scenario, BL showed approximately 2% higher p at the plateau than GBLUP. The discrepancy in p between selection criteria declined as the number of QTL increased. GS (GBLUP and BL) moved the favourable alleles towards fixation faster than BLUP, and selection on BLUP showed faster fixation than PS as shown in Figure 1.

The discrepancy in changes in allele frequencies, ∆p, between selection criteria also reduced as the number of QTL increased (Figure 2A, B and C). In the *4QTL_h5* scenario, BL performed better than GBLUP in most of the generations. Phenotypic selection showed the lowest ∆p until G_13_. After G_13,_ the results were no longer comparable since most QTL were fixed and resulted in a large standard error in ∆p. For the other scenarios, ∆p was stable and PS resulted in a lower ∆p compared to all other criteria.

Genetic variance across 25 generations was affected by the number of QTL controlling the trait (Figure 2D, E and F). All selection criteria showed a faster initial increase, a higher peak and a faster final loss in genetic variance when the number of QTL was lower. The faster initial increase in variance was due to a rapid rise in p given an easier identification of animals with a favourable combination of alleles when the number of QTL was small. The higher peak with a smaller number of QTL resulted from all QTL reaching intermediate allele frequencies at the same time with few QTL, while with more QTL it took more generations for all p to move past 0.5 (Figure 1). The loss in genetic variance occurred due to the rapid fixation of favourable alleles. In all presented scenarios, the genetic variance for PS initially showed a slower increase and subsequently reached a higher peak and decreased more slowly compared to other selection criteria, followed by BLUP and BL. GBLUP showed the lowest peak and the most significant decay in genetic variance.

The pattern of accuracy was also influenced by the number of QTL for a given heritability (Figure 2G, H and I). Accuracy in a given generation depended on the genetic variance that was maintained. Similar to genetic variance, all selection criteria also showed a faster initial gain, a higher peak and a faster final loss in the accuracy when the number of QTL was lower. In the *4QTL_h5* scenario, BL showed a higher peak in accuracy (0.72) relative to other criteria, including GBLUP (0.67). After the peak, the decay in accuracy for BL and GBLUP was greater than for other criteria and accuracy became lower than accuracy for PS after G_13_ due to fixation of favourable QTL alleles. In the *40QTL_h5* and the *100QTL_h5* scenarios, BL showed a slightly higher accuracy than GBLUP after six and eight generations, respectively, partly due to a higher genetic variance.

### Loss of favourable QTL alleles

**Figure 3:**
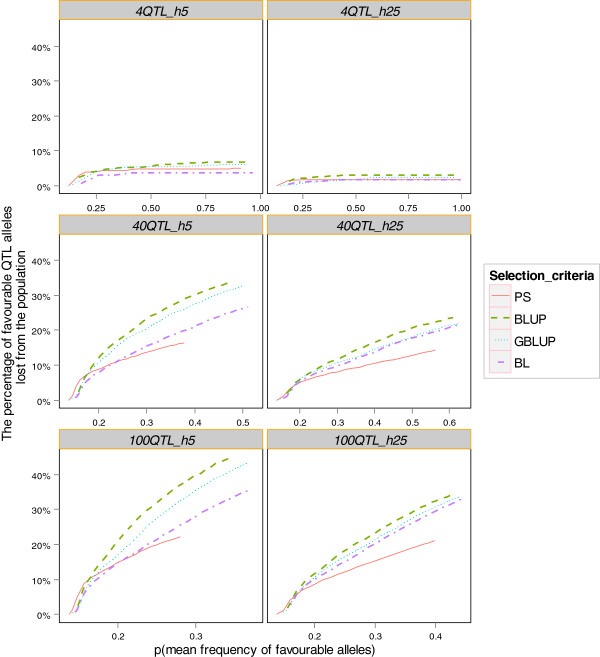
The percentage of favourable QTL alleles lost from the population plotted against the mean frequency of favourable alleles for each generation.

The loss of favourable QTL alleles was significantly influenced by the number of QTL and heritability (Figure 3). Generally, the number of favourable alleles lost was greater when the number of QTL was higher. For instance, in the *4QTL_h25* scenario, where each QTL had a larger effect, less than 5% of the favourable alleles were lost after 25 generations for all selection criteria. This loss increased to 21% for PS and to 35% for BLUP in the *100QTL_h25* scenario. It was also found that to reach the same average level of p, a lower heritability led to a greater loss of favourable alleles during the process. Moreover, for all selection criteria and scenarios, a greater loss of favourable alleles occurred in the first few generations and this slowed down thereafter.

For the *4QTL* scenarios, BL performed the best among the selection criteria in terms of maintaining favourable QTL, while BLUP performed the worst. For the other scenarios, BLUP was still distinguished from the other selection criteria by having on average the highest loss of favourable alleles. Bayesian Lasso showed an advantage for both maintaining more favourable alleles and increasing the average p. This result could partly explain why BL had a higher final average frequency of favourable alleles and maintained more genetic variance than GBLUP. For PS, however, its low improvement in average frequency of favourable alleles compared to all other criteria was compensated by it having the smallest loss of favourable alleles. The difference in the loss of favourable alleles between BLUP, GBLUP and BL became smaller at a higher heritability. In addition, the loss of rare favourable alleles followed the pattern for all favourable alleles (results not shown).

### Hitch-hiking

In presenting the hitch-hiking effect, only the generations in which QTL were not yet fixed were considered because linked loci would no longer have a hitch-hiking effect if the QTL was fixed [10]. The first QTL was fixed by generation 8 for the most extreme scenarios, i.e. with BLUP, GBLUP and BL in the *4QTL_h25* scenario. Thus, allele frequency changes for LN in the first eight generations were used for analysis in order to allow systematic comparisons for all selection criteria and all scenarios.

**Figure 4:**
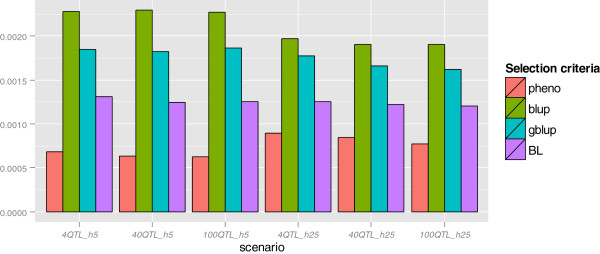
Variance of allele frequency changes at selectively neutral loci located on chromosome 5 (SN).

Figure 4 provides information on *σ*^2^(Δ*p*_*SN*_) for different selection criteria for all scenarios. Heritability had an impact on *σ*^2^(Δ*p*_*SN*_) but the number of QTL did not. The value of *σ*^2^(Δ*p*_*SN*_) was reduced with a higher heritability for BLUP, GBLUP and BL, regardless of the number of QTL. However, with PS, *σ*^2^(Δ*p*_*SN*_) increased with heritability. For all scenarios, BLUP showed the highest *σ*^2^(Δ*p*_*SN*_), followed by GBLUP, BL and PS.

**Figure 5:**
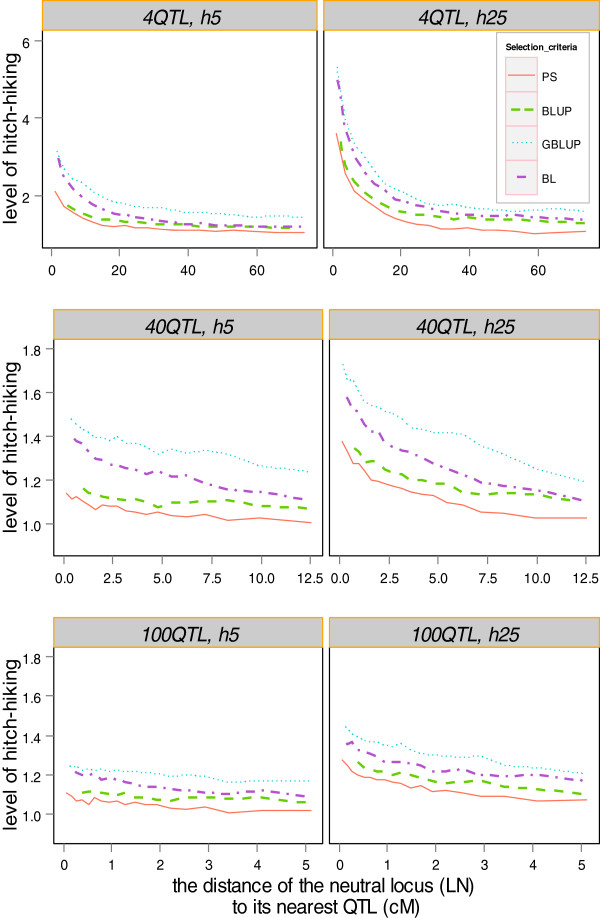
**The hitch-hiking effect.** The hitch-hiking effect was presented as the variance of allele frequency changes at linked loci relative to the variance of allele frequency changes at unlinked neutral loci, as a function of distance from the QTL (in cM); allele frequency changes of all linked loci were calculated and sorted by their distance in cM to the closest QTL; subsequently we performed a sliding window of 40 000 records of variance of allele frequency changes at each linked locus; note that the scale of both axes differ for scenarios with different numbers of QTL.

Figure 5 illustrates the relationship between the hitch-hiking effect and the distance between LN and its nearest QTL and shows a distinct peak in the level of hitch-hiking in the vicinity of the QTL for all scenarios and for all selection criteria. This means that a linkage drag existed around the selected loci, even when the accuracy of selection and the allele substitution effect were relatively low. The amount of hitch-hiking declined as the distance of the LN to a QTL increased.

The amount of hitch-hiking across the entire genome was affected by the number of QTL and heritability (Figure 5). In general, a higher heritability and a lower number of QTL (i.e. a higher allele substitution effect) could result in a stronger hitch-hiking effect surrounding the QTL. For all scenarios, GBLUP showed the largest hitch-hiking effect, followed by BL, BLUP and PS. For example, the ratio of *σ*^2^(Δ*p*_*LN*_) to *σ*^2^(Δ*p*_*SN*_) at the peak ranged from 5.51 for GBLUP and from 5.28 for PS in the *4QTL_h25* scenarios to 1.28 for GBLUP and 1.11 for PS in *100QTL_h5*. For GBLUP, BL and BLUP, *σ*^2^(Δ*p*_*LN*_) was significantly higher than *σ*^2^(Δ*p*_*SN*_) across the entire genome for the *4QTL_h5* scenario, which implies that even at a distance of more than 75 cM, selection at the QTL dragged blocks of the chromosome more than at random. For PS, in the *4QTL* scenarios, selection at the QTL only dragged a block of less than 50 cM. In the other scenarios, the order of selection criteria according to the level of hitch-hiking was the same.

### Inbreeding

Comparisons of rates of inbreeding were also made based on the first eight generations (Table 2). Results showed that ∆F_ped_ was significantly influenced by heritability for BLUP, GBLUP and PS. A higher heritability resulted in a reduction in ∆F_ped_ for BLUP and GBLUP but an increase in ∆F_ped_ for PS. While increasing the number of QTL decreased ∆F_ped_ in most cases, this effect was not significant. BLUP showed higher pedigree inbreeding than all other selection criteria, followed by GBLUP, BL and finally PS. For the *4QTL_h5* scenario for instance, ∆F_ped_ was 9% higher for BLUP than for GBLUP.

**Table 2:** **The rate of inbreeding based on pedigree (∆F**_**ped **_**(±SE), %) and runs of homozygosity (∆F**_**ROH **_**(±SE), %) and the Mendelian selection differential (Md (±SE))**

		**4 QTL**				**40 QTL**				**100 QTL**			
		**Selection criterion**				**Selection criterion**				**Selection criterion**			
	**h2**	**PS**	**BL**	**GBLUP**	**BLUP**	**PS**	**BL**	**GBLUP**	**BLUP**	**PS**	**BL**	**GBLUP**	**BLUP**
∆F_ped*_	0.05	0.70	1.32	1.87	2.04	0.69	1.30	1.85	2.08	0.67	1.30	1.88	2.01
		(0.02)	(0.04)	(0.05)	(0.05)	(0.02)	(0.04)	(0.05)	(0.05)	(0.02)	(0.04)	(0.05)	(0.04)
	0.25	0.92	1.23	1.56	1.70	0.90	1.20	1.52	1.67	0.88	1.26	1.50	1.65
		(0.03)	(0.04)	(0.04)	(0.05)	(0.02)	(0.04)	(0.04)	(0.05)	(0.03)	(0.03)	(0.03)	(0.04)
∆F_ROH50_^†^	0.05	0.73	1.44	2.18	2.15	0.70	1.44	2.13	2.12	0.68	1.43	2.15	2.05
		(0.01)	(0.04)	(0.04)	(0.04)	(0.01)	(0.03)	(0.03)	(0.04)	(0.01)	(0.03)	(0.04)	(0.03)
	0.25	1.03	1.42	1.92	1.98	0.92	1.41	1.84	1.81	0.89	1.40	1.74	1.77
		(0.03)	(0.03)	(0.04)	(0.04)	(0.02)	(0.03)	(0.03)	(0.03)	(0.02)	(0.03)	(0.03)	(0.03)
Md^§^	0.05	0.06	0.11	0.07	0.03	0.03	0.06	0.05	0.02	0.03	0.05	0.05	0.02
		(0.006)	(0.007)	(0.005)	(0.003)	(0.002)	(0.003)	(0.003)	(0.002)	(0.002)	(0.002)	(0.002)	(0.002)
	0.25	0.28	0.27	0.23	0.13	0.18	0.22	0.20	0.14	0.15	0.18	0.18	0.12
		(0.016)	(0.019)	(0.013)	(0.008)	(0.005)	(0.007)	(0.005)	(0.007)	(0.005)	(0.005)	(0.005)	(0.005)

*The rate of inbreeding was calculated based on the first eight generations; ^†^the number followed by ROH represents the number of markers involved in each window; ^§^mendelian selection differential in generation 8.

The cut-off length for calculating ROH did not have a significant effect on ∆F_ROH_. Thus, only ∆F_ROH50_ was included in Table 2. For all scenarios and all selection criteria, ∆F_ROH50_ was significantly higher than ∆F_ped_, except for PS, for which the difference was not significant for the ≥ *40QTL* scenarios. For all criteria except BL, ∆F_ROH50_ was significantly higher for the *4QTL* scenarios than for the ≥ *40QTL* scenarios. The difference between ∆F_ped_ and ∆F_ROH50_ tended to be smaller with a higher number of QTL. In addition, in any generation, the value of the inbreeding coefficient measured with a cut-off length of 50 SNPs (F_ROH50_) was closer to F_ped_ as compared to F_ROH10_ and F_ROH25_ (results not shown). This result was as expected because F_ROH50_ captures more recent inbreeding within the pedigree.

The ranking of scenarios based ∆F_ROH50_, however, was different from that based on ∆F_ped_, e.g., ∆F_ROH50_ was even higher for GBLUP than for BLUP in the *h5* scenarios. The difference between ∆F_ROH_ and ∆F_ped_ for BL was also greater than for PS, but relatively smaller than for GBLUP. For instance, ∆F_ROH50_ was approximately 10% to 15% higher than ∆F_ped_ for the *4QTL* scenario. This suggests that the rate of inbreeding measured by pedigree does not accurately reflect the rate of true inbreeding for GS.

In G_8_, the average mendelian selection differential at the QTL was lowest for BLUP and highest for BL, except for the *4QTL_h25* scenario. Mendelian selection differential was smaller with a higher number of QTL and a lower heritability, but the difference in mendelian selection differential between the *40QTL_h5* and *100QTL_h5* scenarios was not significant.

## Discussion

### Inbreeding

The results of this study demonstrate that directional selection on favorable alleles can reduce heterozygosity of loci that are closely linked to one or more QTL. The reduction of genetic diversity surrounding the QTL is caused by the effect of “hitch-hiking”, which was first termed by Maynard Smith and Haigh [10]. Our results indicate that with a limited population size, inbreeding is not only caused by random genetic drift but also by direct selection on the QTL. Hitch-hiking due to linkage gradually removed linked neutral polymorphisms from the population, thus also acting as an important mechanism to reduce the genetic diversity and further increase the rate of inbreeding. This mechanism appeared more substantial under GS, which contributes to a large difference between the rate of inbreeding measured by pedigree and by ROH (∆F_ped_ and ∆F_ROH_) for GS. Furthermore, the discrepancy between ∆F_ped_ and ∆F_ROH_ greatly depended on the number of QTL.

### Genetic drift, inbreeding and loss of loci

In the current study, random genetic drift with selection on the different criteria was measured by the variance of gene frequency changes for selectively neutral loci, *σ*^2^(Δ*p*_*SN*_), which were simulated on chromosome 5 (Figure 4). The loci on chromosome 5 were in linkage equilibrium (LE) with the QTL since the QTL were on different chromosomes, so the value of *σ*^2^(Δ*p*_*SN*_) reflects the impact of genetic drift due to the emphasis on selection of families. The results showed that at lower heritability, i.e. 0.05, genetic drift was more pronounced for GS and BLUP because the emphasis on sib information in these selection criteria was high, so co-selection of relatives increased as the heritability decreased, in contrast to PS [6]. The results also showed that GBLUP led to greater genetic drift, more loss of favorable alleles and higher inbreeding than BL. A possible explanation is that, compared to BL, GBLUP is more affected by family relationships among individuals, which is similar to traditional BLUP. Habier et al. [30] conducted a simulation study in which all markers were in LE with 10 QTL and showed that, with sufficiently dense markers, the accuracy of GEBV from GBLUP was only marginally smaller than the accuracy from BLUP, which suggested that GBLUP also puts substantial emphasis on genetic relationships. Bayesian methods, however, captured much less genetic relationship than GBLUP with dense markers [30]. Table 2 also showed that BL is less sensitive, with regard to inbreeding and loss of favorable alleles, to the heritability than GBLUP, which indicates that it is less sensitive to family relationships. The current study also showed that in most scenarios, BL resulted in a higher mendelian selection differential than GBLUP and BLUP, indicating that BL had a greater ability to capture the within-family differentiation.

The greater genetic drift with GBLUP led to a larger chance of losing favorable alleles and greater pedigree inbreeding, as shown in Figure 3 and Table 2. A greater loss of favorable alleles from GBLUP than from BL may also be attributed to the different weight on the rare alleles for prediction. In the current study, the assumption that all QTL had equal variance in the simulation resulted in rare alleles to have larger allele substitution effects. GBLUP results in greater shrinkage towards zero for the effects of markers that have a low minor allele frequency, even though they had large effects [31]. The alleles at these markers will therefore have a larger risk of being lost and contribute to inbreeding with GBLUP compared to BL. Another important finding was that more favorable alleles were lost when the number of QTL was greater, likely because selection pressure on each QTL is smaller and therefore, drift becomes relatively more important.

Inbreeding results from drift because alleles become IBD. In fact, the variance of the change in allele frequency at a locus in one generation is *σ*^2^(Δ*p*) ≅ *F*_*IBD*_ * *p*_0_ * (1 − *p*_0_), where *F*_*IBD*_ is the inbreeding coefficient measured by IBD [2]. Provided that changes in allele frequencies were adjusted by their standard deviation, the variance of changes in allele frequencies serves as a good indicator of inbreeding. Inbreeding predicted from pedigree, ∆F_ped_, assumes that all alleles are selectively neutral, which was valid for the markers on chromosome 5. Therefore, the conclusion from results of ∆F_ped_ was consistent with results obtained from *σ*^2^(Δ*p*_*SN*_), that is, ∆F_ped_ increased with *σ*^2^(Δ*p*_*SN*_).

In the current study, the level of hitch-hiking was measured by the ratio of *σ*^2^(Δ*p*_*LN*_) to *σ*^2^(Δ*p*_*SN*_). Based on this ratio being greater than 1, Figure 5 indicates that linked neutral loci yielded a higher IBD than neutral loci, in particular for loci in the vicinity of QTL. This also implies that the genetic variance at a QTL can be explained by loci (markers) near the QTL and under the condition that a higher LD exists between the QTL and the adjacent loci, as opposed to by loci that are more distant.

### Hitch-hiking

Hitch-hiking can be considerable if the QTL effect is large. For example, Pedersen et al. [13] suggested that the hitch-hiking effect of positive selection on a single QTL with a large effect can span up to 1 Morgan, which is consistent with our findings for BLUP and GS but does not hold for PS, for which the hitch-hiking did not impact drift on the entire chromosome. Moreover, when a trait is affected by more QTL, a significant hitch-hiking occurred around each QTL, but the proportion of the genome involved in hitch-hiking was reduced. A higher accuracy of any selection criterion due to a higher heritability caused a higher peak and steeper slope of hitch-hiking. The most likely reasons for these findings are that, first, the selection pressure for the QTL is stronger with a higher accuracy, and second, a higher accuracy leads to faster fixation of the QTL and thus LD between adjacent loci will be broken down with a more rapid speed relative to a lower accuracy. This implies that strength of selection on the QTL may be an essential factor for the level of hitch-hiking observed for each selection criteria. This is consistent with the findings of Kaplan et al. [32], who developed a model for hitch-hiking and stated that in regions of low crossing-over, strongly selected substitutions in the history of the sample can substantially reduce the number of polymorphic sites in a random sample of genes compared to that expected under a neutral model.

Our results show that hitch-hiking was greater with GS due to higher accuracy of selection on the QTL, as stated above. Another reason might be that instead of directly selecting the QTL, selection acts on markers in LD with the QTL, which results in more IBD as well as larger ROH segments across the genome. Figure 5 shows that the hitch-hiking was more marked with GBLUP than with BL, probably because of the assumption of GBLUP that all markers contribute equally to the observed variation. Habier et al. [30] reported that, with 1000 markers in LD with 10 QTL, GBLUP fitted 100% of SNPs when predicting GEBV, while only a small subset of markers (1.82% to 5.23%) were fitted in Bayesian methods. In our study, BL provides an example to illustrate in terms of an a priori distribution in which each marker was weighted differently, so that a limited number of markers were used to capture the QTL. With a few QTL, the assumption for BL is more appropriate than for GBLUP. BL was able to identify the position of a large QTL and only a few SNPs near the QTL were required for prediction, whereas in GBLUP, the effect of a QTL was spread over a larger number of markers. Therefore, with a limited number of QTL the IBD peaks were lower with BL than with GBLUP, leading to a lower overall hitch-hiking and genomic inbreeding, as seen from ∆F_ROH_ (Table 2). If the genetic model resembles the polygenic model, this conclusion might not hold. For instance, based on a simulation using 1000 QTL, Sonesson et al. [12] found that the Bayesian method resulted in higher genomic inbreeding than GBLUP. However, in agreement with our study, Sonesson et al. [12] found that under truncation selection, genomic inbreeding was substantially greater than pedigree inbreeding, especially with GS.

### Genetic variance and genetic trends

The genetic variance maintained over generations differed between the BLUP and GS scenarios in two aspects: in BLUP, fixation of QTL was slower and genetic drift was more severe due to increased co-selection of relatives as parents. For GS, the loss of favourable alleles was attributed to genetic drift as well as to low LD between QTL and markers. For GBLUP, fixation of QTL seemed to outweigh genetic drift, in particular with a small number of QTL, resulting in a faster reduction in the genetic variance than with more QTL. Moreover, our results indicate that with a limited number of QTL affecting the trait, BL ensured a larger long-term response, as shown by the favourable allele frequencies, due to the fact that BL maintained more genetic variance.

### Other scenarios

It should be noted that several aspects of the simulation lack realism and might affect the results. First only a limited number of QTL were simulated but in reality most of the traits, e.g. human height, are likely to be polygenic. Pedigree inbreeding might be a good estimate of true inbreeding under the infinitesimal model, because the discrepancy between pedigree and true inbreeding over all QTL decreases with the number of QTL [33]. Therefore, it would be interesting to investigate the hitch-hiking effect with much more QTL in further studies, since the suggestion that pedigree inbreeding serves as a good estimator of true inbreeding is difficult to verity from the current results. The second limitation was that all QTL were simulated to explain equal variance in order to maximize the effective number of QTL. However, in reality, QTL effects will show more variability [34]. The difference in the loss of favourable alleles between GBLUP and BL might be smaller if the QTL effects followed a gamma distribution, since GBLUP is expected to lose fewer rare alleles than BL. However, with the assumption of equal variance, the allelic effects of markers were more similar to each other, so that the hitch-hiking is similar across the QTL. Moreover, if the effects of QTL are too different, it will also be difficult to see the pattern of hitch-hiking on the basis of the distance of any QTL to all its linked loci. Another limitation is that the initial favourable allele frequencies were considered to be smaller than 0.3. The explanations for this choice are that: first, the purpose of the study was to observe the hitch-hiking effects of the QTL. If the QTL become fixed rapidly, the linked loci will no longer experience hitch-hiking. This will happen within a few generations when the number of QTL is low, which will generate less replicates for hitch-hiking results and also make it difficult to compare scenarios. Second, the whole process of the change in gene frequencies can be observed if the initial frequencies for the favourable alleles are lower. Third, favourable alleles with lower initial frequencies are expected to have a larger chance of being lost, which was of interest in this study. To test whether the initial allele frequencies affected the final conclusions, the simulation was also run using QTL with minor allele frequencies greater than 0.01 (See Additional file 1: Figure S1). In this scenario, the difference in the favourable allele frequencies in any generation is smaller compared to the scenario in which the favourable allele has a lower initial frequency, especially at a higher heritability. It was also shown that the ranking of methods based on the loss of favourable alleles was not greatly affected by the initial frequencies of favourable QTL alleles but, in general, the loss was substantially reduced with a higher initial frequency (See Additional file 2: Figure S2).

### Implication

In our study, we did not take differences in recombination rates between sexes into account [35]. In addition, recombinations were sampled from a Poisson distribution and were randomly placed along the chromosome assuming a uniform distribution, but in reality, recombination patterns are rarely uniform across the (human) genome [36]. Non-uniformity of recombination rates (hot spots and cold spots) along a chromosome can have an impact on the pattern of LD, e.g. LD blocks. It is unclear how recombination patterns would affect the result of hitch-hiking, but previous results have shown that positive selection can result in a distinctive footprint that can extend across very large segments, even in regions with high recombination rates [37]. Moreover, we only simulated a single trait for each scenario, and the closely linked loci did not affect any other trait. In reality, the closely linked loci might be deleterious mutations that negatively affect a trait. For example, Chun et al. [38] reported that, in humans, within genomic regions that show evidence of hitch-hiking by adaptive substitutions, there were fewer neutral but a similar number of deleterious SNPs compared to other genomic regions. They also found that disease alleles within hitch-hiking regions can cause auto-immune disorders, cancers and mental disorders. This implies that for animals, positive selection on traits of interest could potentially increase the frequencies of linked deleterious alleles. Therefore, the footprint of GS must be taken into account. Sonesson et al. [12] used optimum contribution selection and showed that this method can spread the selection pressure quite evenly over many loci in order to control the increase in overall IBD. Another method would be to weight marker effects by the inverse of their allele frequencies, as suggested by Goddard [39], such that specific emphasis on the QTL with a large effect would be avoided. Then, the selection intensity can be desirably spread across the genomic regions, which can reduce the footprint of selection and maximise long-term genetic gain [39].

## Conclusions

In conclusion, signatures of selection play an important role in the variation observed at the genome-wide level. Neutral variation can be shaped to a great extent by hitch-hiking effects that are associated with selection, rather than just by genetic drift. The hitch-hiking effect is a key factor that leads to large differences between pedigree inbreeding and genomic inbreeding, especially with genomic selection. When inbreeding was measured by pedigree information, selection on genomic BLUP EBV resulted in lower levels of inbreeding than selection on phenotype BLUP EBV, but this did not always apply when inbreeding was measured by runs of homozygosity. Bayesian Lasso was found to result in less genetic drift, less loss of favorable alleles and less pedigree and genomic inbreeding when the number of QTL was up to 100. When implementing long-term genomic selection, genomic control of inbreeding is essential to reduce the considerable hitch-hiking effects that are associated with genomic selection, regardless of the prediction model used.

## Competing interests

The authors declare that they have no competing interests.

## Authors’ contributions

HL wrote the simulation computer program and drafted the manuscript. PB, ACS and THEM conceived and designed the study and edited the drafted manuscript. All authors have read and approved the final manuscript.

## Supplementary Material

Additional file 1 **Favourable QTL allele frequencies (p) across 25 generations.** Identical to Figure 1, but the initial allele frequencies of all favourable alleles range from 0.01 to 0.99. Click here for file

Additional file 2 **The percentage of favourable QTL alleles lost from the population plotted against the mean frequency of favourable alleles for each generation.** Identical to Figure 3, but the initial allele frequencies of all favourable alleles range from 0.01 to 0.99. Click here for file
