# Supplementary figures and images for: Allele frequency changes due to hitch-hiking in genomic selection programs
Source: Genet Sel Evol. 2014 Feb 4;46(1):8. doi: 10.1186/1297-9686-46-8 (PMC3942192; doi:10.1186/1297-9686-46-8)

## Slide 1
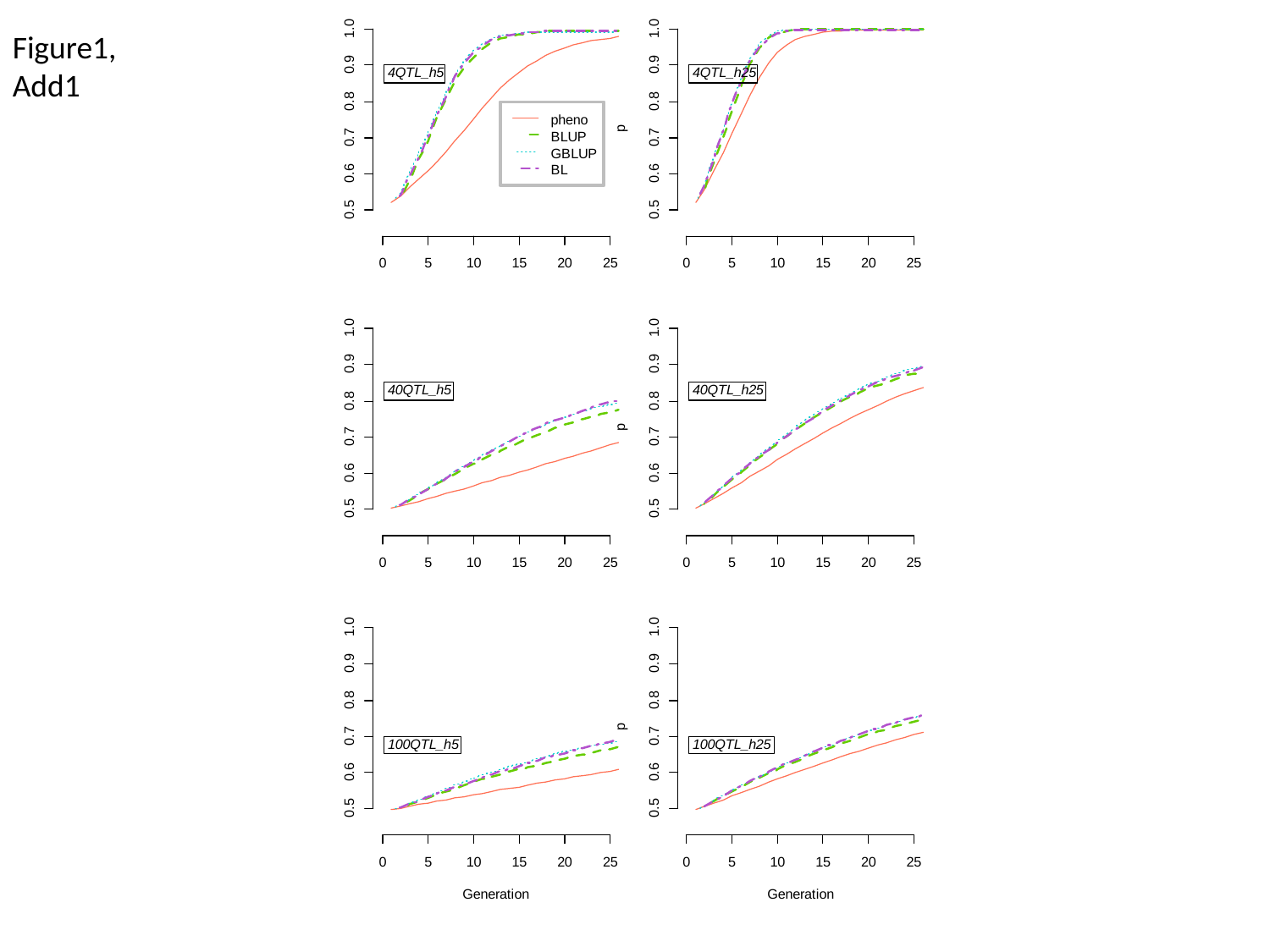

Figure1, Add1

Supplement: Additional file 1 — Favourable QTL allele frequencies (p) across 25 generations. Identical to Figure 1, but the initial allele frequencies of all favourable alleles range from 0.01 to 0.99. [file 1297-9686-46-8-S1.pptx]

## Slide 1
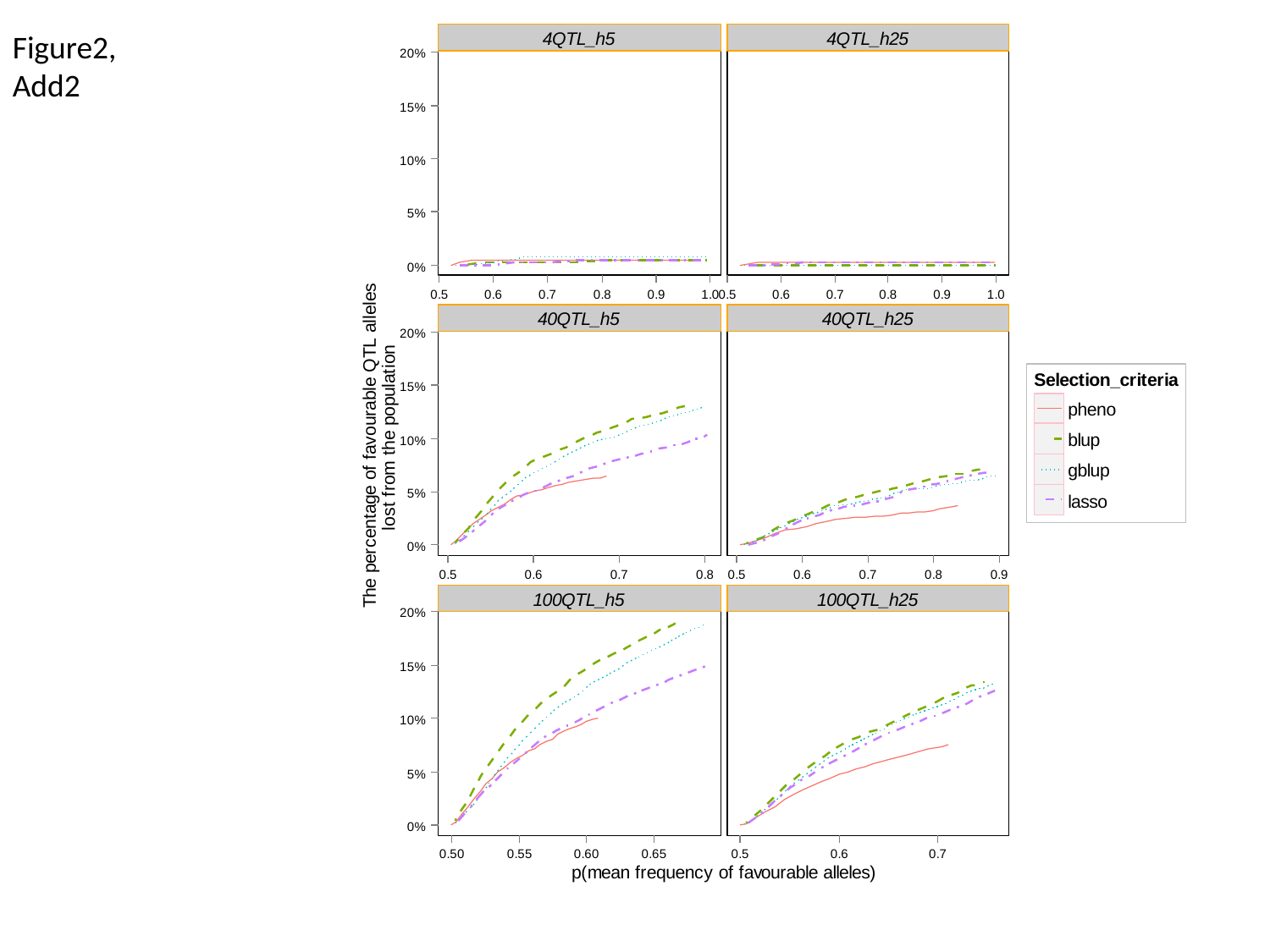

Figure2, Add2

Supplement: Additional file 2 — The percentage of favourable QTL alleles lost from the population plotted against the mean frequency of favourable alleles for each generation. Identical to Figure 3, but the initial allele frequencies of all favourable alleles range from 0.01 to 0.99. [file 1297-9686-46-8-S2.pptx]
